# Secondary brain abscess following simple renal cyst infection: a case report

**DOI:** 10.1186/1471-2377-14-130

**Published:** 2014-06-16

**Authors:** Nobuhiro Akuzawa, Tenshi Osawa, Masayuki Totsuka, Takashi Hatori, Kunihiko Imai, Yonosuke Kitahara, Masahiko Kurabayashi

**Affiliations:** 1 Departments of Internal Medicine, 1-7-13 Koun-cho, Maebashi, Gunma 371-0025, Japan; 2 Neurology, Gunma Chuo Hospital, 1-7-13 Koun-cho, Maebashi, Gunma 371-0025, Japan; 3 Department of Medicine and Biological Science, Graduate School of Medicine, Gunma University, 3-39-22 Showa-machi, Maebashi, Gunma 371-8511, Japan

**Keywords:** Bacteremia, Brain abscess, *Escherichia coli*, Simple renal cyst

## Abstract

**Background:**

*Escherichia coli (E. coli)* is the most common causative bacteria of neonatal meningitis, but hematogenous intracranial *E. coli* infection is rare in adults. Moreover, intracranial abscess formation owing to *E. coli*, including brain abscesses and subdural empyema formation, is extremely rare. We herein present a case involving a patient with a brain abscess owing to *E. coli* following a simple renal cyst infection. A review of the literature is also presented.

**Case presentation:**

A 77-year-old Japanese woman with a history of polymyalgia rheumatica was admitted to our hospital because of persistent fever, right flank pain, and pyuria. Intravenous antibiotics were administered; however, her level of consciousness deteriorated 6 days after admission. Contrast-enhanced magnetic resonance imaging showed a brain abscess in the left occipital lobe and pyogenic ventriculitis. Enhanced abdominal computed tomography revealed a right renal cyst with heterogeneous content. Culture of urine, blood, and aspirated pus from the infected cyst revealed *E. coli* with identical antibiotic sensitivity in all sites, suggesting that the cyst infection and subsequent bacteremia might have caused the brain abscess. The patient recovered after a 6-week course of meropenem.

**Conclusion:**

The prognosis of patients with *E. coli*-associated intracranial abscess is usually poor. Advanced age and immunosuppression may be potent risk factors for intracranial abscess formation owing to the hematogenous spread of *E. coli.*

## Background

Although brain abscesses are relatively uncommon, they remain potentially fatal central nervous system (CNS) infections despite the evolution of neurosurgical techniques, new antibiotics, and new imaging technologies. Brain abscesses are usually caused by contiguous infections such as sinusitis or middle ear infections; however, hematogenous spread of pyogenic pathogens from remote-organ infections can also cause brain abscesses [1].

The clinical signs of brain abscesses are nonspecific. The most common symptoms are reportedly headache and fever, but the classical triad of fever, headache, and nausea is seen in up to 20% of patients [2]. Focal neurological deficits are reportedly recognized in 57% of patients [2]. Additionally, laboratory findings may show a normal white blood cell (WBC) count or C-reactive protein (CRP) level [2]. Thus, early imaging studies including enhanced brain computed tomography (CT) or magnetic resonance imaging (MRI) are of cardinal importance for a definitive diagnosis and appropriate treatment in the early period [1,2].

We herein present a rare adult case of a left occipital lobe brain abscess that likely resulted from the hematogenous spread of an *Escherichia coli* (*E. coli*) right renal cyst infection followed by formation of pyogenic intraventricular empyema.

## Case presentation

A 77-year-old Japanese woman was admitted to our hospital because of fever of unknown origin. She had developed general fatigue and a slight fever 2 weeks before admission. Headache and a fever higher than 38°C had developed 3 days before admission, and oral cefditoren pivoxil prescribed at another hospital had been ineffective. She had a 2-year history of polymyalgia rheumatica and chronic gastritis. She had initially been treated with 15 mg/day of prednisolone for the polymyalgia rheumatica. Her dosage of prednisolone was tapered with improvement of her symptoms, and she had been treated with prednisolone (5 mg/day) and famotidine (20 mg/day) for 1 year before admission. She had no other medical or family history and did not smoke or drink alcohol. Physical examination on admission revealed a height of 156 cm, weight of 49 kg, temperature of 40.2°C, heart rate of 120 beats/min, and blood pressure of 116/62 mmHg. No obvious abnormalities of the chest or abdomen were found with the exception of slight right flank pain. Neurological examination also showed normal findings; her Glasgow Coma Scale (GCS) score was maximal at 15 (E4V5M6). She was ambulatory; no symptoms suggesting agnosia, agraphia, or any other higher brain dysfunction were observed on admission. In addition, finger perimetry showed no obvious visual field deficits. Laboratory findings showed a high WBC count (20,200/mm^3^), platelet count (40.4 × 10^4^/mm^3^), and CRP level (10.02 mg/dl). Chest X-ray, abdominal X-ray, and electrocardiographic findings were normal. Urinalysis revealed pyuria with markedly increased WBCs and gram-negative bacilli, suggesting a urinary tract infection. Intravenous administration of ampicillin (6 g/day) was begun immediately after admission. On day 4, her body temperature remained higher than 38°C despite improvement in the WBC count (18,300/mm^3^) and CRP level (3.91 mg/dl). On the same day, *E. coli* was revealed in urine and blood culture specimens taken on admission. Although the *E. coli* was sensitive to ampicillin, we substituted ceftriaxone (2 g/day) for ampicillin on day 4 based on the results of the antibiotic sensitivity test.

**Figure 1:**
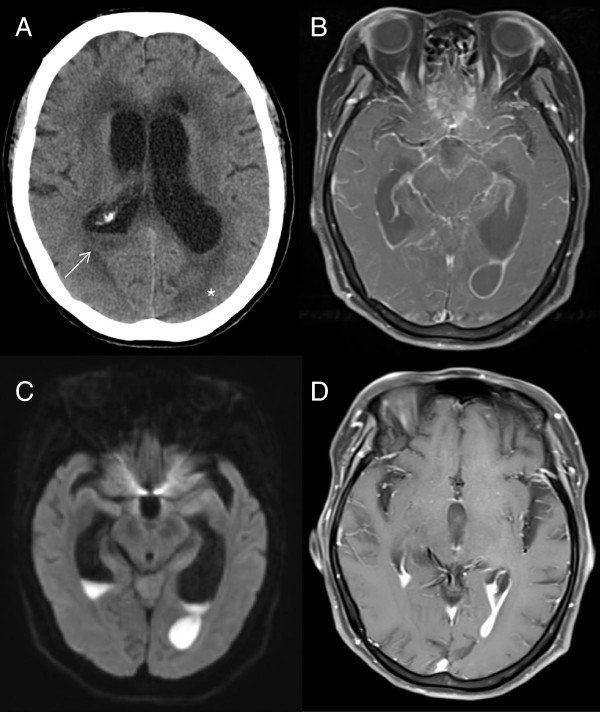
**Plain computed tomography (CT) and contrast-enhanced magnetic resonance imaging (MRI) of the brain on days 6 and 30. A**: Plain CT on day 6. Debris in the right lateral ventricle (white arrow) and a space-occupying lesion with surrounding edema in the left occipital lobe (asterisk) were observed. **B**: T1-weighted, contrast-enhanced MRI on day 6. The left occipital lesion displayed ring-like enhancement. **C**: Diffusion-weighted imaging on day 6. Homogenous high-intensity signaling of the lesion was observed. **D**: Contrast-enhanced T1-weighted imaging on day 30. The left occipital lesion dramatically decreased in size.

**Figure 2:**
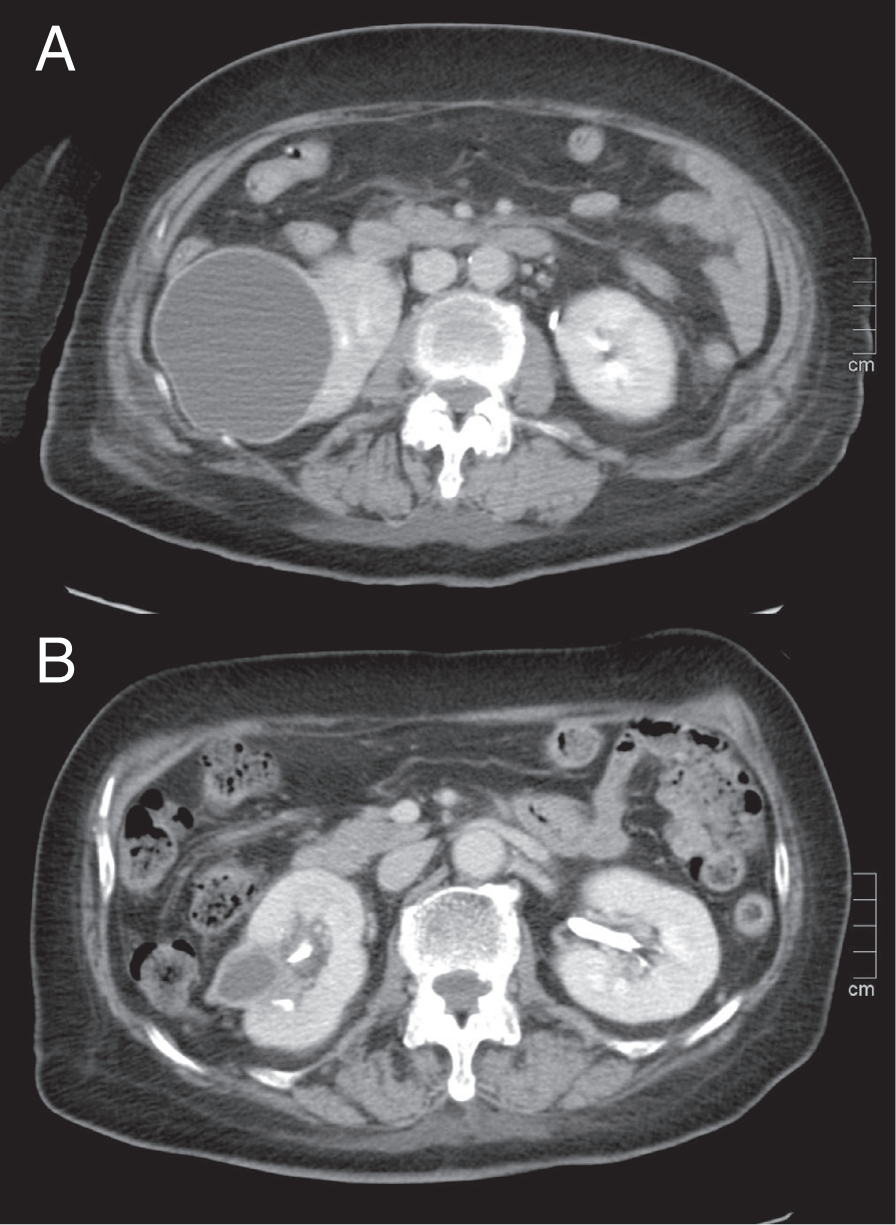
**Contrast-enhanced computed tomography (CT) images of the abdomen on days 6 and 35. A**: CT image on day 6 showed a right renal cyst (9.3 × 7.2 cm). The CT attenuation value of the inner cyst contents was about 25 Hounsfield units, much higher than the value of water. The lateral margin of the cyst was faintly contrasted. **B**: CT image on day 35. The right renal cyst had markedly decreased in size after the drainage procedure.

On day 5, her body temperature dropped to 37.5°C. However, she began to drop her spoon frequently during meals and complain of difficulty in donning her shirt. On day 6, she complained of difficulty in seeing. Although she did not exhibit severe palsy or ataxia, she could no longer eat a meal, change her clothes, or walk by herself without nursing care. She was considerably confused, and it was difficult to evaluate her neurological function precisely. Her level of consciousness sharply deteriorated thereafter, and her GCS score dropped to 9 (E2V3M4). Her WBC count and CRP level on day 6 were 15,600/mm^3^ and 3.63 mg/dl, respectively. Electrolyte, thyroid hormone, and blood glucose levels were normal. Plain brain CT on day 6 showed debris in the right lateral ventricle and a tumor-like lesion in the left posterior lobe (Figure 1A). Contrast-enhanced MRI on day 6 revealed a tumor-like lesion in the left posterior lobe with ring-like enhancement on T1-weighted imaging (Figure 1B). On diffusion-weighted imaging, this lesion also showed homogenous high intensity, which seemed to be connected with the left lateral ventricle, and obvious debris was present in both lateral ventriculi (Figure 1C). Based on these findings, we considered that ventricular rupture of a brain abscess was the most probable cause of her deterioration. The size of the abscess was 1.6 × 1.8 cm. Subsequent abdominal contrast-enhanced CT on day 6 showed a unilocular right renal cyst with heterogeneous and high-density content, suggesting a renal cyst infection (Figure 2A). Abdominal ultrasonographic findings were inconclusive for a cyst infection. Contrast-enhanced chest CT showed no remarkable findings. Further laboratory and transthoracic echocardiographic diagnostics excluded potential risk factors for brain abscess, such as human immunodeficiency virus infection, fungal infection, endocarditis, and pulmonary hypertension. We immediately consulted with neurosurgeons and decided to manage the right occipital lesion with intravenous meropenem administration (6 g/day). Her consciousness gradually improved thereafter; her GCS score recovered to 12 (E3V4M5) on day 9. She was slightly confused and showed impaired orientation, but her ability to converse with us had improved. Brain MRI on day 9 revealed no remarkable changes in the size of the abscess. No signs of high intracranial pressure or hydrocephalus were present either clinically or radiographically. Cerebrospinal fluid (CSF) analysis on day 9 revealed a slight increase in leukocytes (64/mm^3^), including 23/mm^3^ (36%) lymphocytes and 41/mm^3^ (64%) neutrophils with no atypical cells, as well as high total protein (517 mg/dl) and low glucose (21 mg/dl) levels. Microscopy of CSF smear samples with methylene blue staining, India ink capsule staining, and Gram’s staining detected no bacteria or fungi. The CSF culture was sterile. On day 12, her body temperature remained below 37.5°C, but her WBC count (14,900/mm^3^) and CRP level (4.01 mg/dl) remained high. Additionally, her right flank pain persisted despite antibiotic therapy. MRI on day 12 showed no deterioration of the brain abscess. Based on these findings, we concluded that CT-guided drainage was needed to manage the renal cyst infection. The drainage procedure was performed on day 13, and *E. coli* from the aspirated pus showed the same sensitivity to antibiotics as did the *E. coli* from the blood and urine on admission. The patient showed good progress thereafter. The drainage catheter was removed on day 20. Her GCS score recovered to 15 on day 22. On day 30, she showed almost normal activities of daily life, although she still experienced difficulty in seeing. Her WBC count and CRP level normalized, and enhanced brain MRI showed a dramatic decrease in the abscess size (Figure 1D). Moreover, abdominal enhanced CT on day 35 showed that the right renal cyst had decreased in size (Figure 2B). Formal perimetry was performed by an ophthalmologist on day 35 and showed obvious right inferior quadrantic hemianopsia. Moreover, transesophageal echocardiography using contrast medium on day 35 showed no cryptic abnormalities, including intracardiac right-to-left shunting or small vegetations. Intravenous administration of meropenem was terminated on day 42, and no deterioration was observed even in the absence of oral antibiotics. Following rehabilitation, she was discharged from the hospital on day 60 with right inferior quadrantic hemianopsia as an aftereffect. She has been followed up for 6 months since discharge, and no recurrence of the brain abscess or renal cyst infection has been observed.

## Discussion

The development of a brain abscess owing to hematogenous spread of pathogens from a distant infectious focus, such as a lung abscess, empyema, skin infection, or intra-abdominal infection, is seen in 15–30% of cases [1]. In patients with a cryptogenic brain abscess, examination for potential cardiovascular diseases may reveal congenital heart diseases, patent foramen ovale, or arteriovenous fistula that can permit pathogenic bacteria to bypass the lungs and enter the systemic circulation [1]. Urinary tract infections may cause metastatic brain abscesses, and *Enterobacteriaceae* and *Pseudomonas* species have been reported as causative pathogens [1,2]. In general, however, aerobic and anaerobic streptococci are the most common pathogens in brain abscesses owing to contiguous and hematogenous spread of infection; the frequency of gram-negative bacterial infection is low [1,2]. Basically, *E. coli* and Group B *Streptococcus* are known to be common causative bacteria of neonatal meningitis [3]. Moreover, to the best of our knowledge, only nine adults with *E. coli* intracranial abscesses, including brain abscesses or subdural empyema, have been reported in the past 20 years (Table 1) [4-12]. Additionally, only two cases of *E. coli* brain abscess have been reported [6,9]. Interestingly, eight of the nine reported cases involved older men, implying that age and sex may be potent risk factors. Six patients died within 1 month after diagnosis [4-6,8,10,12]. In five cases, extracranial infection had been recognized prior to the onset, suggesting that the hematogenous spread of *E. coli* might be a pivotal cause of intracranial abscess formation [4,5,7,9,11]. Preceding urinary tract infection was reported in three cases [4,7,11], and only one patient had a history of taking immunosuppressants (corticosteroids) [6]. These reported patients include one with a history of splenectomy [8], one with esophageal cancer undergoing radiochemotherapy [11], and two with diabetes [6,10]. These findings suggest that in addition to sex and age, immunosuppression may be another risk factor for intracranial *E. coli* infection. In our case, the *E. coli* strains obtained from urine, blood, and aspirated pus from the infected renal cyst showed the same minimum inhibitory concentration, suggesting a strong involvement of hematogenous infection. However, one limitation of our report was the inability to determine the genetic identity of these *E. coli* strains by analysis of restriction products after digestion of chromosomal deoxyribonucleic acid with restriction endonuclease.

**Table 1:** **Reported adult cases of brain abscess or subdural empyema owing to *Escherichia coli *** **infection**

**Author**	**Year**	**Age sex**	**Immuno-suppressants**	**Underlying diseases**	**Preceding infection opportunity**	**Lesion**	**Surgical treatment**	**Outcome**
Bakker [4]	1995	88 W	(−)	Column fracture	Orthopedic surgery (hip), UTI	SE	Drainage	Dead
Hirano [5]	1995	86 M	(−)	Chronic cholecystitis	Cholecystitis	SE	Drainage	Dead
Rickert [6]	2000	52 M	Cortico- steroids	DCM, DM	(−)	BA, Malaloplakia	Craniotomy	Dead
Nishi [7]	2005	76 M	(−)	ADPKD	Renal cyst infection	SE	Drainage	Alive
Bachmeyer [8]	2005	55 M	(−)	Esophageal cancer	(−)	SE	(−)	Dead
Doepp [9]	2006	67 M	(−)	PFO	Perianal abscess	BA	(−)	Alive
Adamides [10]	2007	91 M	(−)	Chronic subdural hematoma, DM	Surgical aspiration of subdural hematoma	SE	Drainage	Dead
Narita [11]	2009	80 M	(−)	Post-gastrectomy and splenectomy	Orthopedic surgery (leg, spine), UTI	SE	Drainage	Alive
Redhu [12]	2011	48 M	(−)	(−)	(−)	SE, Pneumo-cephalus	Craniotomy	Dead

ADPKD: autosomal-dominant polycystic kidney disease, DCM: dilated cardiomyopathy, DM: diabetes mellitus, PFO: patent foramen ovale, UTI: urinary tract infection, BA: brain abscess, SE: subdural empyema.

Recent experimental hematogenous meningitis models have indicated that the primary site of entry of circulating *E. coli* into the CNS is the cerebral vasculature, not the choroid plexus [3]. Nevertheless, hematogenous brain abscess formation owing to *E. coli* infection is rare. Bakker *et al.*[4] reported that autopsy of a patient with *E. coli*-induced subdural empyema showed no obvious inflammation in the brain parenchyma. In our reviewed cases, seven of nine patients showed subdural empyema. These findings suggest the presence of key mechanisms preventing *E. coli* infection in the brain parenchyma. In an in vitro blood–brain barrier model using human brain microvascular endothelial cells (HBMECs), *E. coli* was shown to invade and internalize the HBMECs as membrane-bound vacuoles with no changes in the integrity of the HBMEC monolayer [3]. Moreover, *E. coli* enters the CNS with no changes in the blood–brain barrier permeability and no concomitant presence of host inflammatory cells [4]. Once *E. coli* invades the brain parenchyma, microglia, the resident macrophage population in the CNS, may play a key role in recognizing and eliminating the microbes via Toll-like receptors or phagocytic receptors [13]. Additionally, activated microglia produce various pro-inflammatory cytokines, leading to the activation and chemotaxis of peripheral immune cells; however, their phagocytic or killing activity toward microbes is less potent than that of polymorphonuclear leukocytes [13]. A recent study showed that microglia and astrocytes are specifically activated soon after bacterial invasion into the CNS parenchyma [13]. The above findings indicate that impaired glial cell function or an impaired immune response induced by glial cells may contribute to *E. coli* infection in the CNS parenchyma.

Notably, a preceding *E. coli* infection of a simple right renal cyst might have caused the bacteremia and subsequent brain abscess in the present case. Simple renal cysts are usually observed as unilateral and solitary lesions, and the prevalence rate ranges from 7 to 10%, increasing with age [14]. Major complications of simple renal cysts, such as hemorrhage, infection, and rupture, are rare events seen in only 2–4% of affected patients [14]. Suwabe *et al.*[15] showed that renal cysts with high intensity, a fluid–fluid level, or wall thickening on diffusion-weighted imaging suggest the presence of a cyst infection in patients with autosomal dominant polycystic kidney disease. A heterogeneous internal cyst density with no enhancement on CT also suggests a cyst infection [15]. Symptoms of cyst infection are nonspecific. Especially in patients with autosomal-dominant polycystic kidney disease, the most conspicuous symptom is fever; in general, abdominal pain and frank hematuria are not observed [15]. Cyst puncture and aspiration can be diagnostic and may circumvent the need for surgical procedures such as nephrectomy [14]. Physicians should know that cyst infections may cause serious complications, even in patients with simple renal cysts.

## Conclusion

We experienced a rare case of a brain abscess following a simple *E. coli* renal cyst infection. Brain abscess formation owing to hematogenous spread of *E. coli* is very rare. Simple renal cyst infection is also rare, but can cause serious complications. Immunosuppression may contribute to the onset of intracranial *E. coli* abscesses, such as brain abscesses and subdural empyema. Advanced age may also be a potent risk factor.

## Consent

Written informed consent was obtained from the patient for publication of this case report and any accompanying images. A copy of the written consent is available for review by the Editor of this journal.

## Abbreviations

CNS: Central nervous system; CRP: C-reactive protein; CSF: Cerebrospinal fluid; CT: Computed tomography; *E. coli*: *Escherichia coli*; GCS: Glasgow coma scale; HBMEC: Human brain microvascular endothelial cell; MRI: Magnetic resonance imaging; WBC: White blood cell.

## Competing interests

The authors declare that they have no competing interests.

## Authors’ contributions

NA drafted the manuscript. TO, MT, and TH collected the patient data and monitored the patient throughout the whole follow-up period. KI and YK edited the manuscript. MK participated in the study design and coordination and helped to draft the manuscript. All authors have read and approved the final manuscript.

## Pre-publication history

The pre-publication history for this paper can be accessed here:

http://www.biomedcentral.com/1471-2377/14/130/prepub
